# Diagnostic value of [^18^F]AlF-NOTA-FAPI-04 PET/CT in breast cancer: comparison with conventional imaging

**DOI:** 10.3389/fonc.2026.1795128

**Published:** 2026-04-28

**Authors:** Jingru Liu, Yizhen Pang, Tianxin Liu, Shengnan Xu, Jinli Pei, Shijie Wang, Yanluan Guo, Jingjing Wang, Jinming Yu, Wanhu Li, Jie Liu

**Affiliations:** 1 Department of Oncology, Renmin Hospital of Wuhan University, Wuhan, China; 2 Center for Nuclear Medicine and Molecular Imaging, Shandong Cancer Hospital and Institute, Shandong First Medical University and Shandong Academy of Medical Sciences, Jinan, China; 3 Department of Pathology, Shandong Cancer Hospital and Institute, Shandong First Medical University and Shandong Academy of Medical Sciences, Jinan, China

**Keywords:** [^18^F]AlF-NOTA-FAPI-04, breast cancer, conventional imaging, diagnostic accuracy, positron emission tomography/computed tomography (PET/CT)

## Abstract

**Background:**

Conventional imaging has limitations in differentiating benign from malignant breast lesions and in staging breast cancer (BC). This study compared the diagnostic and staging performance of [^18^F]AlF-NOTA-FAPI-04 PET/CT with conventional imaging in suspected breast lesions.

**Methods:**

This single-center *post hoc* retrospective analysis included therapy-naïve participants with suspected breast lesions who underwent [^18^F]AlF-NOTA-FAPI-04 PET/CT between July 2023 and February 2025. All patients underwent ultrasonography, mammography, breast MRI, whole-body CT, and bone scan. Conventional imaging results were based on consensus visual interpretation integrating these modalities. PET/CT images were assessed visually and semi-quantitatively to assess the diagnostic accuracy. Lesions were confirmed by histopathology and follow-up (median, 4.5 months). Diagnostic performance for primary breast lesions, regional lymph nodes metastases (LNM), and distant metastases was evaluated on lesion-based, whereas visual scoring and staging (the AJCC eighth edition) comparisons were patient-based. Receiver operating characteristic/Delong and McNemar χ² analyses were performed using SPSS Statistics 29.0. A two-sided *P* < 0.05 was statistically significant.

**Results:**

Sixty-nine women (median age, 50 years) with 81 breast lesions, 797 regional lymph nodes, and 94 distant metastases were included, and all 69 patients underwent ultrasonography, mammography, breast MRI, whole-body CT, and bone scan. For primary breast lesions, conventional imaging showed sensitivity and specificity of 93.75% and 100.00%, whereas visual PET/CT showed 98.44% and 70.59%. Although LBR_breast_ achieved the highest area under the curve (AUC), its AUC was not significantly different from that of conventional imaging (1.000 vs. 0.970, *P* = 0.078). In contrast, visual PET/CT showed a significantly lower AUC than conventional imaging (0.845 vs. 0.970, *P* = 0.034). For regional LNM detection, visual PET/CT achieved sensitivity and specificity of 95.95% and 97.60%, which were significantly higher than the corresponding values of conventional imaging (73.99% and 95.41%, all *P* < 0.05). PET/CT detected all 94 distant metastases, and patient-based visual scores favored PET/CT over conventional imaging (52 vs. 34). PET/CT also improved the accuracy in predicting pathological N staging (91.43% vs. 65.71%, *P* = 0.008) and final staging (74.29% vs. 45.71%, *P* = 0.009).

**Conclusions:**

[^18^F]AlF-NOTA-FAPI-04 PET/CT outperforms conventional imaging in detecting regional LNM and improving pathological staging accuracy in BC.

## 1 Introduction

For women, breast cancer (BC) is the most commonly diagnosed cancer, accounting for 23.8% of all new cancer cases, and the leading cause of cancer-related death, accounting for 15.4% of all cancer-related death (1). Accurate initial diagnosis, detection of metastases, and precise staging are essential for optimal perioperative treatment decisions. Breast conventional imaging, including ultrasonography, mammography, magnetic resonance imaging (MRI), computed tomography (CT), bone scan and [^18^F]FDG positron emission tomography/computed tomography (PET/CT) are commonly used for diagnosing and staging BC.

Conventional imaging is widely recommended by several guidelines and commonly used in BC diagnosis, but it has limitations (2, 3). In distinguishing between benign breast lesions (BBL) and BC, conventional imaging only provides Breast Imaging-Reporting and Data System (BI-RADS) categories and malignancy risk (4). Moreover, ultrasonography has only 51% sensitivity for detecting axillary lymph node metastasis (LNM), which is lower (20-30%) for mammography (5, 6). These low sensitivity may result in under-staging of the disease and delayed treatment decisions. Although MRI can improve sensitivity, it is not universally feasible for all patients due to factors like metal implants, claustrophobia, long procedure times, and limited availability in primary care. And completing all conventional imaging will prolong the waiting time for surgery. [^18^F]FDG PET/CT, while common used, shows high false-negative rates for small (<1 cm) and low-grade tumors and high false-positive rates for certain benign lesions (7, 8). The sensitivity and specificity to detect LNM were 49% (95% CI: 39–59%) and 94% (95% CI: 91–96%) for [^18^F]FDG PET/CT (5). Consequently, the routine use of [^18^F]FDG PET/CT is not recommended for BC diagnosis and staging of clinical stage I, II, or operable III (9). Beyond [^18^F]FDG, several emerging molecular and immuno-PET tracers have been explored for BC imaging, including agents targeting estrogen receptors and HER2 (10, 11). These tracers provide valuable biologic information and may improve lesion detection in selected molecular subtypes. However, their clinical utility is often limited by subtype specificity, intra-tumoral heterogeneity, and relative slow pharmacokinetics of antibody. Therefore, a more broadly applicable imaging strategy that is less dependent on tumor cell receptor status remains needed. The new improved noninvasive imaging might enhance diagnosis and staging accuracy in BC patients.

Fibroblast activation protein (FAP) is a typical marker of cancer-associated fibroblasts. It shows high expression in malignant cells of BC. While fibroadenomas, breast adenosis, and normal breast epithelial cells rarely or did not express FAP. Malignant BC cells in metastatic lymph nodes can also exhibited significantly elevated FAP expression (12). Therefore, radiolabeled FAP inhibitor (FAPI) can serve as a novel radiotracer and is not dependent on blood glucose levels, unlike routine [^18^F]FDG PET/CT. FAPI-04 exhibits a more robust FAP binding capacity and favorable pharmacokinetics, compared with FAPI-02 and so on (13). FAPI agents are not FDA approved in the U.S. and are considered investigational. Nowadays, researchers have demonstrated success of [^68^Ga]Ga-FAPI-04 PET/CT in diagnosing and staging BC (14, 15). However, [^68^Ga]Ga-FAPI-04 has the disadvantages of a short half-life (68 min) and expensive transport costs (16). [^18^F]F the most popular radioisotope, has a longer half-life (109.7 min), higher resolution, and higher yield synthesis methods (17). [^18^F]AlF-NOTA-FAPI-04 PET/CT has been explored in lung cancer (18), digestive system cancers (19), and glioblastoma (20). It was proven safe and offered high specificity and high contrast for FAP imaging (21). In the field of BC, [^18^F]AlF-NOTA-FAPI-04 PET/CT was proved a potential tool for noninvasive identification of different subtypes (22), but the diagnostic efficacy of [^18^F]AlF-NOTA-FAPI-04 PET/CT has not previously been well established in BC.

The aim of this *post hoc* retrospective analysis of a prospective study was to compare [^18^F]AlF-NOTA-FAPI-04 PET/CT and conventional imaging for the diagnostic and staging performance in differentiating BBL and BC, benign lymph nodes (BLN) and LNM. Additionally, the study explored the diagnostic performance of [^18^F]AlF-NOTA-FAPI-04 PET/CT in identifying distant metastases and relationship between PET/CT parameters and clinicopathologic characteristics in primary lesions.

## 2 Materials and methods

This is a single-center *post hoc* retrospective analysis of a prospective study that has been given ethical approval by the affiliated Cancer Hospital of Shandong First Medical University (institutional review board approval no. SDZLEC2021–112–02). This study complied with the Declaration of Helsinki. All participants provided written informed consents. The necessary ethical and regulatory approvals for the use of FAPI-04 were obtained prior to the study.

### 2.1 Participants

**Figure 1:**
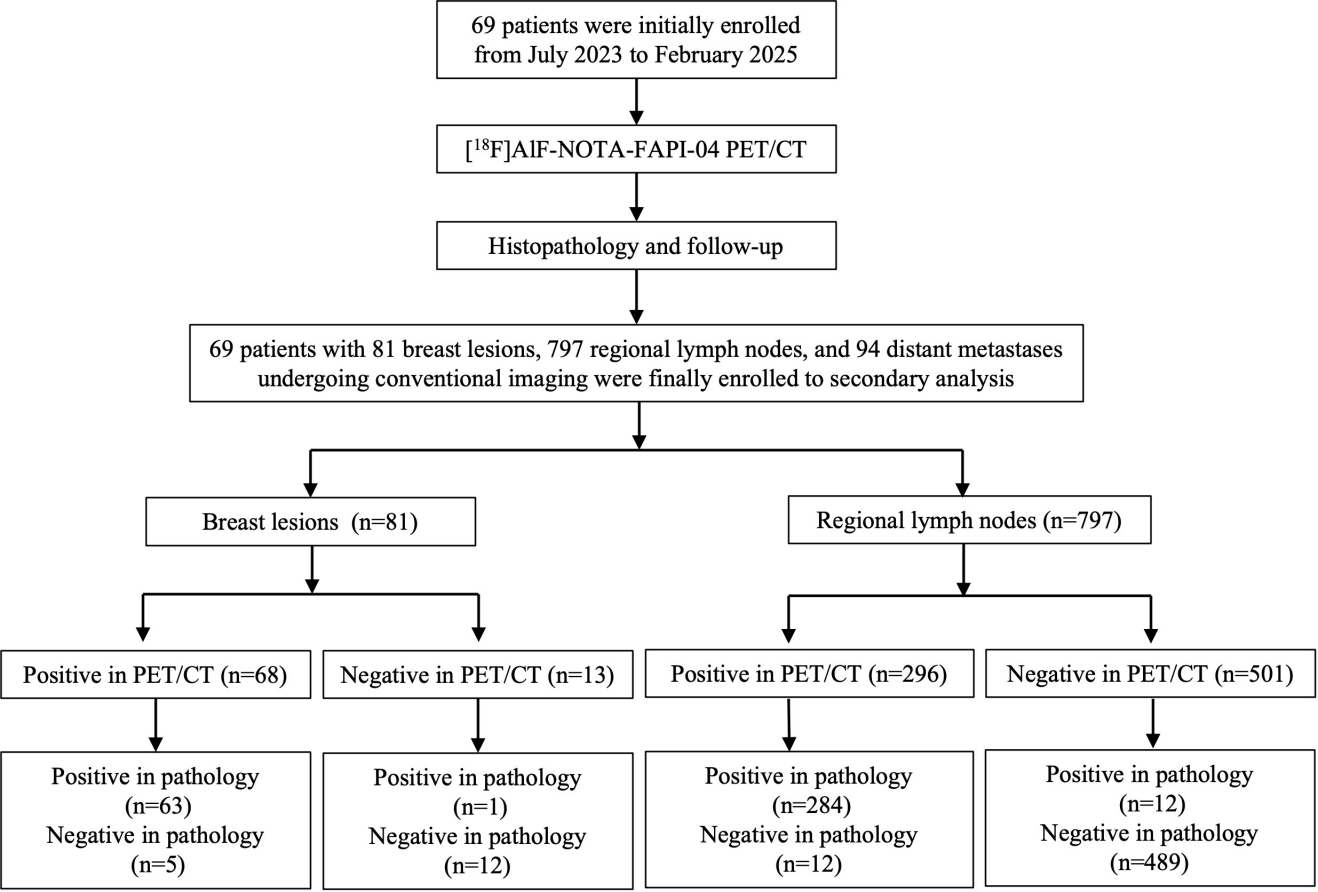
The flow chart of this study.

From July 2023 to February 2025, patients with suspected breast lesions (BI-RADS categories 3, 4, and 5, regardless of lesion size) were enrolled to receive [^18^F]AlF-NOTA-FAPI-04 PET/CT scans at Shandong Cancer Hospital and Institute. The *post hoc* analysis inclusion criteria were: (1) age ≥ 18 years; (2) patients with breast lesion who received digital breast mammography, breast ultrasound, breast MRI, whole-body CT, and whole-body bone scan; (3) breast lesions histopathologic examination obtained from biopsy or surgery; (4) [^18^F]AlF-NOTA-FAPI-04 PET/CT scan performed before any antitumor treatment, biopsy or surgery. Exclusion criteria were: (1) conventional imaging incompletion; (2) other primary tumors; (3) any antitumor treatment before PET/CT scan. The flow chart is presented in **Figure 1**.

### 2.2 [^18^F]AlF-NOTA-FAPI-04 PET/CT scanning and image acquisition

The procedure of [^18^F]AlF-NOTA-FAPI-04 preparation was the same as the previous report (23), NOTA-FAPI-04 (Nanchang Tanzhen Biotechnology Co., Ltd., Jiangxi, China) was radiolabeled with [^18^F]AlF.

Regardless of blood glucose levels and whether or not fasting, patients were injected with [^18^F]AlF-NOTA-FAPI-04 at 4.81 MBq/kg (0.12 mCi/kg) intravenously. After approximately one hour, patients underwent scanning from the top of the skull to the midthigh with an integrated in-line PET/CT system (Gemini TF Big Bore; Philips Healthcare, Cleveland, OH, USA). Parameters of low-dose CT scanning without contrast were as follows: 300 mAs, 120 kV, 512 × 512 matrix, rotation time of 1.0 s, pitch index of 0.688, layer thickness of 2 mm, CT dose index of 8.7 mGy, and dose-length product of 901.3 mGy·cm. After CT scan acquisition, PET scanning was immediately performed (in 3-dimensional mode with a 200 × 200 matrix, each bed position imaging time of 1 minute). Then, the data was reconstructed (Body-ctac-SB. Lstcln, Biograph three-dimensional iterative reconstruction software [version 3.6.2], time-of-flight correction) after random, attenuation, and scatter correction. The PET images, CT images, and fused PET/CT images were displayed on a nuclear medical workstation (Beijing Mozi Healthcare Technology Co., Ltd., Beijing, China) as coronal, sagittal, and axial sections.

### 2.3 [^18^F]AlF-NOTA-FAPI-04 PET/CT image analysis

Two experienced nuclear medicine physicians (J.Y. and Y.G., with 41 and 9 years of experience) were blinded to the results of conventional imaging, histopathologic examinations, and other diagnostic information of all cases. They analyzed images independently and agreed to identify primary BC, regional LNM, and distant metastases. For visual analysis, if the uptake of lesions exceeded that of the adjacent background tissue and was not related to normal physiologic uptake, it was marked as positive. For semiquantitative analysis, semiquantitative parameters (SUV_max_) were automatically measured by a 3-dimensional contouring program with a 30% isocontour. We measured the SUV_mean_ of 1 cm^3^ areas in the descending aorta and contralateral normal breast in the same anatomical quadrant. Then, the ratio of primary breast lesion’s SUV_max_ to SUV_mean_ of normal tissue (blood pool and contralateral breast) was defined as the lesion-to-background ratio (LBR_blood_ and LBR_breast_). The T-stage was determined on fused PET/CT images by measuring the maximum diameter of the primary tumor. Tumor boundaries were automatically delineated using a 3-dimensional contouring program with a 30% isocontour threshold, and the longest tumor diameter within this contour was recorded, integrating both FAPI-04 uptake extent and anatomical size.

### 2.4 Conventional imaging and gold standard

All patients underwent breast ultrasonography, digital breast mammography, breast MRI, whole-body CT, and whole-body bone scan. The breast ultrasonography was performed using a GE Logiq E9 ultrasound machine (GE Healthcare, Chicago, IL, USA) equipped with a high-frequency linear probe (7–12 MHz). The mammography was conducted using a Siemens MAMMOMAT Inspiration system (Siemens Healthcare, Erlangen, Germany). MRI was conducted using a Siemens Magnetom Skyra 3.0T MRI machine (Siemens Healthcare, Erlangen, Germany) with a dedicated breast coil. Two experienced radiologists reached consensus on BI-RADS categories, LNM, and distant metastases on every conventional imaging, blinded to other results. Integrative analysis of conventional imaging refers to a comprehensive consideration of the diagnostic results from ultrasonography, mammography, MRI, CT, and bone scan, where the higher BI-RADS category of the primary lesion is chosen. If either imaging indicates the presence of LNM or distant metastases, it is defined as LNM and distant metastases.

All breast lesions were confirmed by histopathology. The nature of the lymph nodes and distant metastases were confirmed by histopathology (from fine needle aspiration, core needle biopsy, sentinel lymph node biopsy, and axillary dissection) or imaging and clinical follow-up (median follow-up time, 4.5 months [range, 3.0–25.6 weeks]). Like the previous report, for patients whose primary breast lesions were excised directly, experienced breast surgeons simultaneously dissected all suspicious lymph nodes in the surgical field, taking all results from the preoperative conventional imaging and PET/CT into consideration (24). For patients receiving neoadjuvant treatments or those with distant metastases, morphologic and size changes were monitored during systemic treatments. Positive criteria during follow-up were (1) substantial tumor size reduction after antitumor therapy with treatment response, or noticeable enlargement after approximately 6 weeks later without treatment response; (2) presence of new metastatic disease. Negative criteria during follow-up were (1) no substantial change in lesion size after antitumor treatment; (2) negative findings at follow-up imaging. The eighth edition of the AJCC TNM staging system (25) was applied for clinical and pathological staging. Pathological stage analysis was conducted only on patients who underwent surgery directly without receiving neoadjuvant therapy.

### 2.5 Visual scoring system

A visual scoring system, adapted from a previously published study (26), was used to compare the lesion detection capabilities of [^18^F]AlF-NOTA-FAPI-04 PET/CT and conventional imaging. The lesion area (primary tumor and peritoneal metastases) or number (positive lymph nodes, liver, bone, and other tissues metastases) were visually estimated in the same patient by the 2 imaging modalities. If the area/number of lesions detected by PET/CT was > 1 and < 3, 3–5 or > 5 times more than that of conventional imaging, PET/CT was scored 1, 2, and 3, respectively. Conversely, if conventional imaging detected a greater lesion area/number than PET/CT, the corresponding score was assigned in favor of conventional imaging. PET-negative/conventional imaging–positive constellations were included in the conventional imaging–favored category. A score of 0 indicated the same area/number of lesions detected by the 2 imaging modalities.

### 2.6 Date collection and organization

For each patient, demographic and clinical variables were collected, including age, sex, menopausal status, and Karnofsky performance status. Lesion-level variables included the number and type of breast lesions, lymph node status, and distant metastatic status. Imaging variables from conventional imaging included BI-RADS category of the primary lesion, assessment of regional lymph node metastasis, and assessment of distant metastasis. PET/CT variables included visual assessment results and semiquantitative parameters, including SUV_max_, LBR_blood_, and LBR_breast_. Staging-related variables included clinical TNM stage and, where available, pathological TNM stage according to the AJCC 8th edition. Follow-up and reference-standard data, including histopathology and imaging/clinical follow-up results, were also recorded. All data were entered, organized, and checked in Microsoft Excel (Microsoft Corp., Redmond, WA, USA) before statistical analysis in SPSS Statistics 29.0 (IBM Corporation, Armonk, USA). The specific variables were collected and defined according to the methods described in the preceding subsections.

### 2.7 Statistical analysis

Categorical variables were presented as frequency counts (percentages) and compared using chi-square or Wilcoxon rank-sum tests. Continuous variables were expressed as medians (lower and upper quartiles). Normality was assessed according to sample size: the Shapiro-Wilk test was used for small samples (n < 30), and the Kolmogorov-Smirnov test was used for larger samples (n ≥ 30). Normally distributed continuous variables with non-homogeneous variance were compared using the *t’* test, while non-normally distributed continuous variables were compared using the Mann–Whitney *U* test. For diagnostic performance evaluation, analyses of primary breast lesions, regional lymph nodes, and distant metastatic lesions were primarily performed on lesion-based, whereas visual scoring comparisons and staging comparisons were conducted on patient-based. Receiver-operating-characteristic (ROC) curve analysis was employed to evaluate the diagnostic efficacy and determine cut-off values. Diagnostic efficacy was compared using Delong test of the area under the curve (AUC) and McNemar *χ^2^* test with the four-grid table. Based on a two-sided hypothesis, *P* < 0.05 was considered statistically significant. All statistical analyses were conducted using SPSS Statistics 29.0 (IBM Corporation, Armonk, USA).

## 3 Results

### 3.1 Patient characteristics

From July 2023 to February 2025, 69 patients with a total of 81 breast lesions, 797 regional lymph nodes, and 94 distant metastases, were included in this *post hoc* retrospective analysis (**Figure 1**). No adverse events were observed in any of the patients.

**Table 1** summarizes the characteristics of patients in this study. The patients’ ages ranged from 25 to 87 years (median age, 50 years). All patients were female. Twenty-seven patients (39.13%) were postmenopausal. Among the 81 breast lesions, there were 7 lesions of breast fibroadenoma, 10 lesions of breast adenosis, 3 lesions of ductal carcinoma *in situ*, 59 lesions of invasive ductal carcinoma, and 2 lesions of invasive lobular carcinoma. Of the 64 BC in 64 patients, 35 were directly excised, 25 received neoadjuvant treatments, and 4 had distant metastases. A total of 296 regional LNM were confirmed, and 501 regional lymph nodes were BLN. In the advanced patients, there were 23 metastases in mediastinal lymph nodes, 5 in the liver, and 66 in bones. The distribution of clinical TNM stage is also shown in **Table 1**.

**Table 1:** Characteristics in this study.

Characteristic	No. of patients (%)
Total patients	69
Total breast lesions	81
Total regional lymph nodes	797
Total distant metastases	94
Median age in years (range)	50 (25-87)
Menstrual state	
Premenopausal	42 (60.87)
Postmenopausal	27 (39.13)
Karnofsky Performance Status	
100	54 (78.26)
90	15 (21.74)
Histopathology of breast lesions	
Breast fibroadenoma	7 (8.64)
Breast adenosis	10 (12.35)
Ductal carcinoma in situ	3 (3.70)
Invasive ductal carcinoma	59 (72.84)
Invasive lobular carcinoma	2 (2.47)
Molecular subtype based on immunohistochemistry	
Luminal A	12 (18.75)
Luminal B (HER-2 positive)	10 (15.63)
Luminal B (HER-2 negative)	24 (37.50)
HER-2 overexpressing	9 (14.06)
Triple negative like	9 (14.06)
Nottingham grade in invasive carcinoma	
II	41 (67.21)
III	20 (32.79)
Classification of regional lymph nodes	
Benign lymph nodes	501 (62.86)
Lymph nodes metastases	296 (37.14)
Location of distant metastases	
Mediastinal lymph nodes	23 (24.47)
Liver	5 (5.32)
Bone	66 (70.21)
Clinical TNM staging (AJCC eighth edition)	
IA	10 (15.63)
IIA	11 (17.19)
IIB	13 (20.31)
IIIA	9 (14.06)
IIIB	1 (1.56)
IIIC	16 (25.00)
IV	4 (6.25)

### 3.2 Detection of primary tumors

**Figure 2:**
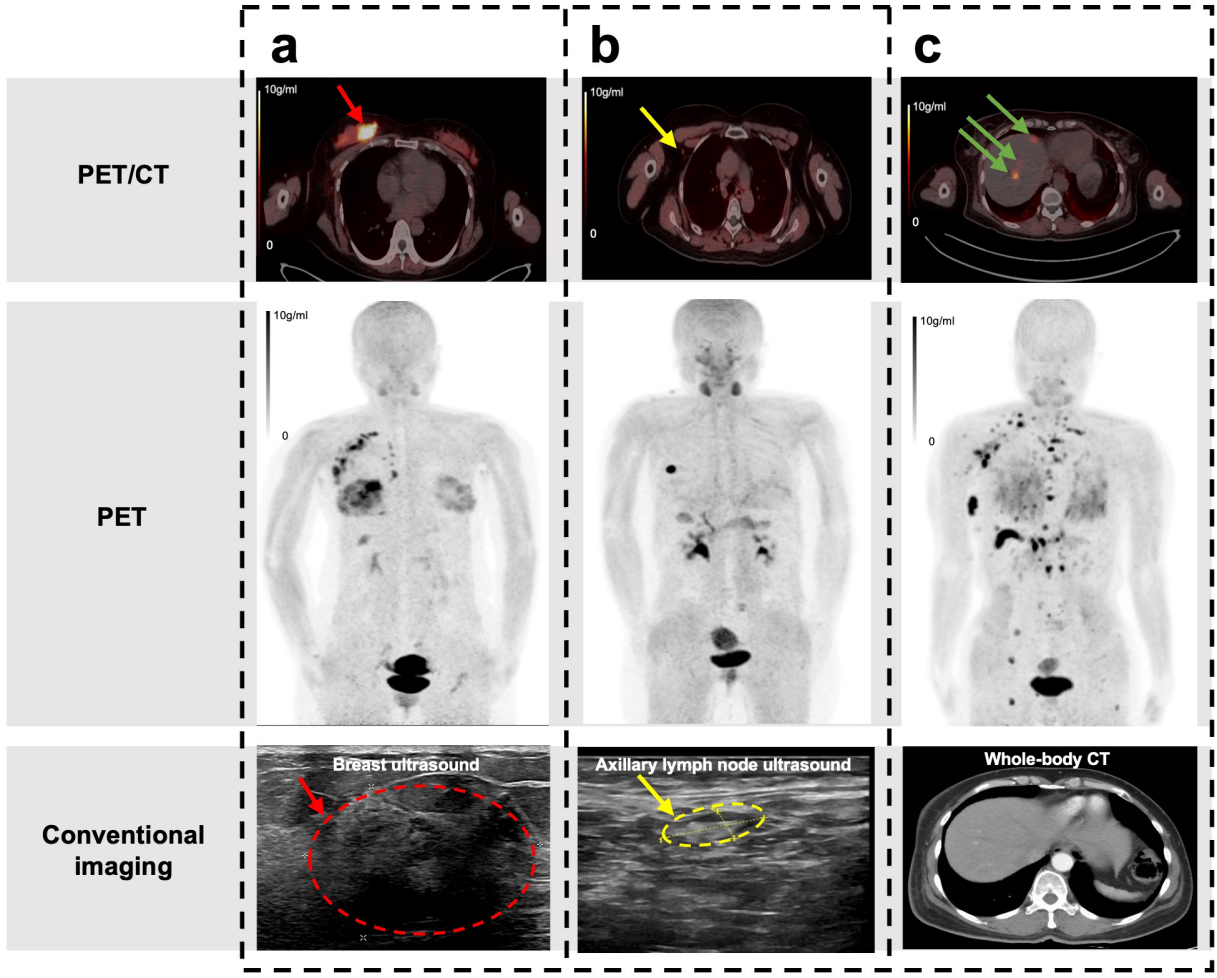
Typical [^18^F]AlF-NOTA-FAPI-04 PET/CT images used to detect breast cancers, regional lymph node metastases, and distant metastases. **(a)** Breast cancer was positive on FAPI PET/CT but classified as BI-RADS category 4a on conventional imaging. **(b)** Lymph node metastasis was detected on FAPI PET/CT but missed on conventional imaging. **(c)** Liver metastases were positive on FAPI PET/CT but not detected on conventional imaging.

All BBLs were classified as BI-RADS category 3 or 4a. In contrast, most BC lesions were classified as BI-RADS category ≥ 4b (93.74%, *P* < 0.001). Particularly, four lesions classified as BI-RADS 3 and 4a were BC (**Table 2**, **Figure 2a**). Through ROC curve analysis, BI-RADS categories 3 and 4a on conventional imaging were identified as BBL, while BI-RADS categories 4b, 4c, and 5 were diagnosed as BC with 93.75% sensitivity, 100% specificity, and 95.06% accuracy (AUC = 0.970, *P* < 0.001) (**Table 3**).

**Table 2:** Comparisons of BI-RADS categories from conventional imaging and [^18^F]AlF-NOTA-FAPI-04 PET/CT semiquantitative parameters between benign breast lesions and breast cancer.

Variables	Benign breast lesions	Invasive ductal carcinomas	*P*
BI-RADS categories			<0.001
3	9 (52.94%)	2 (3.13%)	
4a	8 (47.06%)	2 (3.13%)	
4b	–	4 (6.25%)	
4c	–	19 (29.69%)	
5	–	37 (57.80%)	
SUV_max_	3.15 (2.71 – 4.77)	16.58 (11.16 – 20.91)	<0.001
LBR_blood_	3.50 (2.41– 4.53)	15.08 (10.23 – 20.11)	<0.001
LBR_breast_	2.89 (2.34 - 3.48)	15.81 (10.29 – 25.72)	<0.001

BI-RADS, Breast Imaging-Reporting and Data System; SUV, standardized uptake value; LBR, lesion-to-background ratio.

**Table 3:** ROC quantitative analysis of conventional imaging and [^18^F]AlF-NOTA-FAPI-04 PET/CT in differentiating breast cancer from benign breast lesions.

Variables	Cutoff	AUC (95% CI)	*P*	Sensitivity (%)	Specificity (%)	PPV (%)	NPV (%)	Accuracy (%)
Conventional imaging	4a/4b	0.970 (0.934-1.005)	<0.001	93.75	100.00	100.00	80.95	95.06
Visual analysis	NA	0.845 (0.711-0.979)	<0.001	98.44	70.59	92.65	92.31	92.59
SUV_max_	5.75	0.968 (0.931-1.005)	<0.001	96.88	88.24	96.88	88.24	95.06
LBR_blood_	7.26	0.964 (0.925-1.003)	<0.001	87.50	94.12	98.25	66.67	88.89
LBR_breast_	3.86	1.000 (1.000-1.000)	<0.001	100.00	100.00	100.00	100.00	100.00

ROC, receiver operating characteristic; AUC, area under the curve; PPV, positive predictive value; NPV, negative predictive value; NA, not available; SUV, standardized uptake value; LBR, lesion-to-background ratio.

**Figure 3:**
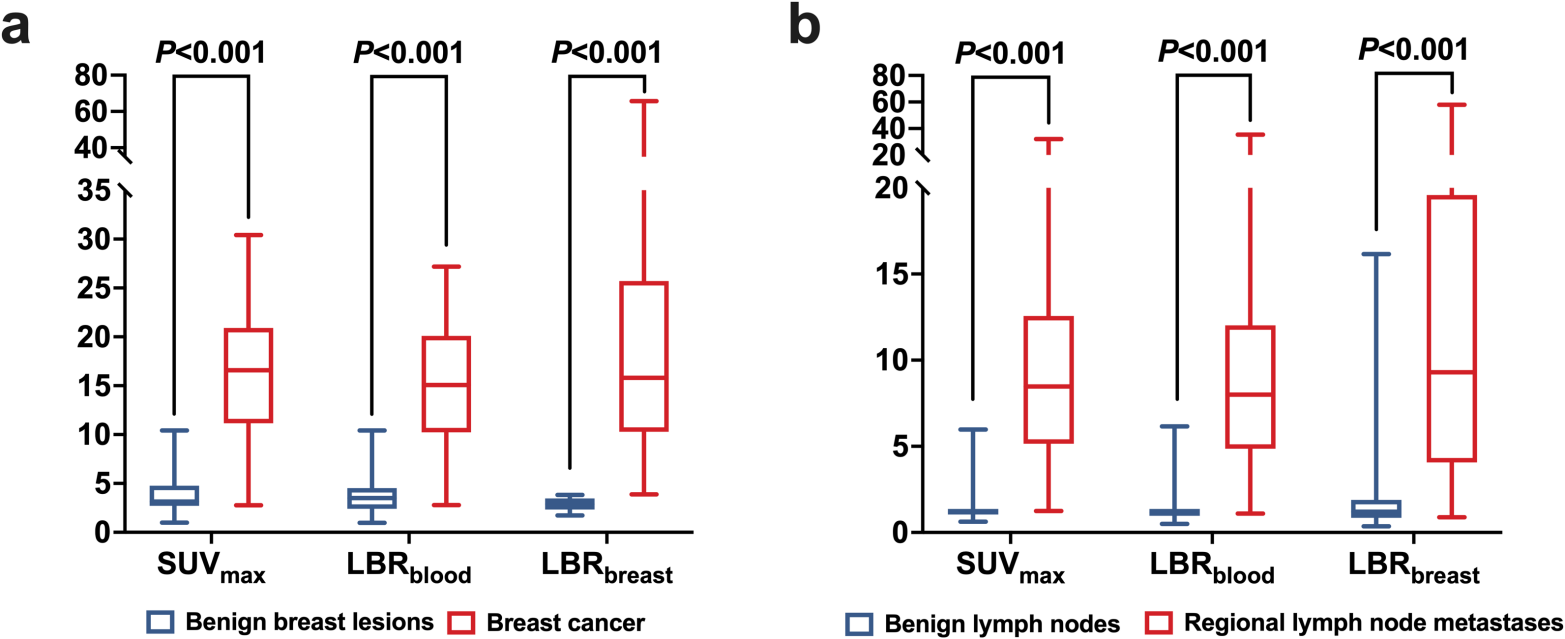
Comparisons of [^18^F]AlF-NOTA-FAPI-04 PET/CT semiquantitative parameters. **(a)** between benign breast lesions and breast cancer, **(b)** between benign lymph nodes and regional lymph node metastases (with *P* value by *t’* test and Mann–Whitney *U* test).

**Figure 4:**
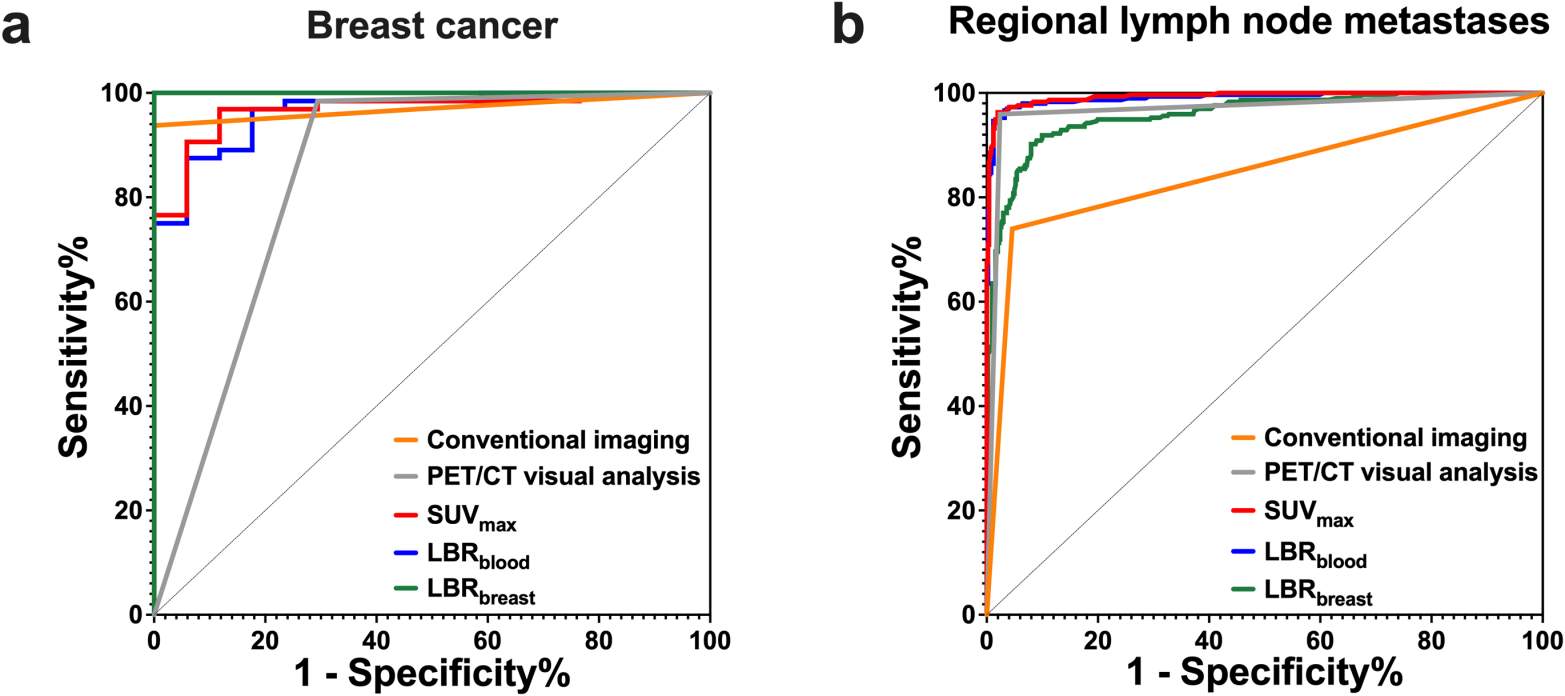
ROC curves of conventional imaging and [^18^F]AlF-NOTA-FAPI-04 PET/CT in differentiating breast cancer from benign breast lesions **(a)**, and regional lymph node metastases from benign lymph nodes **(b)**.

[^18^F]AlF-NOTA-FAPI-04 PET/CT detected 68 positive lesions, including 5 false-positive lesions (2 cases of breast fibroadenoma and 3 cases of breast adenosis). Consequently, the sensitivity and specificity of visual analysis were 98.44% and 70.59% (**Table 3**). Among semiquantitative parameters, SUV and LBR were significantly higher in BC lesions compared with BBLs (*P* < 0.001) (**Table 2**, **Figure 3a**). ROC curve analysis identified all parameters as diagnostic indicators for BC (*P* < 0.001). Although LBR_breast_ achieved the highest AUC of 1.000, its AUC was not significantly different from those of conventional imaging, SUV_max_, or LBR_blood_ (all *P* > 0.05). In contrast, visual analysis of PET/CT showed a significantly lower AUC than conventional imaging, SUV_max_, LBR_blood_, and LBR_breast_ (all *P* < 0.05) (**Table 3**, **Figure 4a**).

### 3.3 Detection of regional LNM

Out of 296 regional LNM, 219 were diagnosed as positive by conventional imaging, resulting in a sensitivity of 73.99%. Conversely, only 23 out of 501 BLN were misdiagnosed as positive (specificity = 95.41%) (**Table 4**).

**Table 4:** Comparisons of conventional imaging and [^18^F]AlF-NOTA-FAPI-04 PET/CT semiquantitative parameters between benign lymph nodes and regional lymph node metastases.

Variables	Benign lymph node	Lymph node metastasis	*P*
Conventional imaging			<0.001
Negative	478 (95.41%)	77 (26.01%)	
Positive	23 (4.59%)	219 (73.99%)	
SUV_max_	1.20 (1.04 – 1.36)	8.46 (5.18 – 12.56)	<0.001
LBR_blood_	1.17 (0.97 – 1.37)	7.93 (4.88 – 12.01)	<0.001
LBR_breast_	1.22 (0.86 – 1.89)	9.30 (4.10 – 19.58)	<0.001

SUV, standardized uptake value; LBR, lesion-to-background ratio.

**Figure 5:**
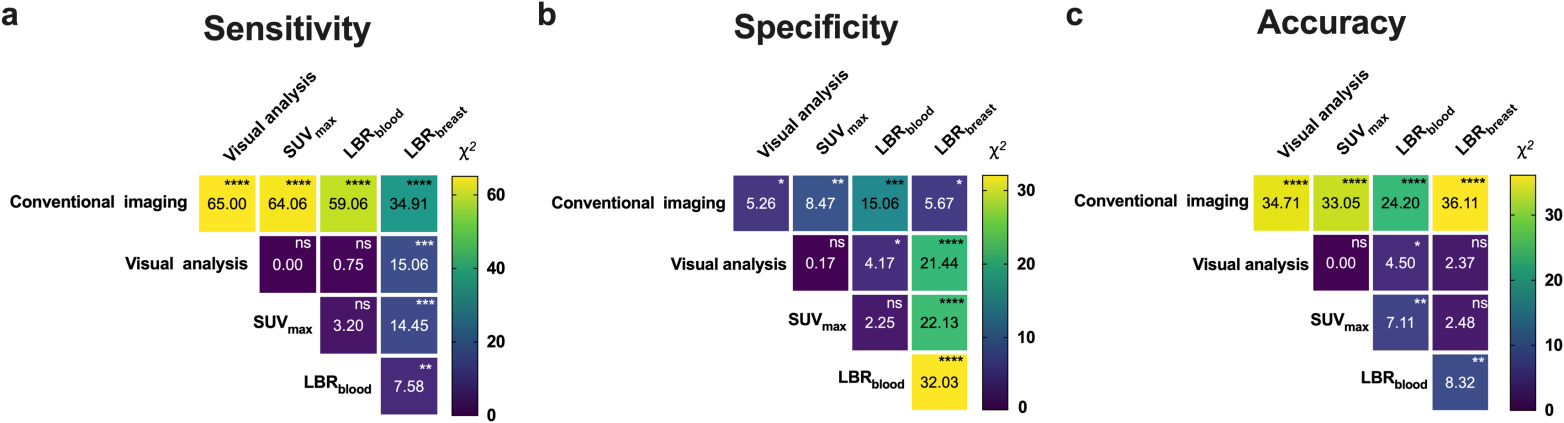
Comparations of diagnostic sensitivity **(a)**, specificity **(b)**, and accuracy **(c)** between conventional imaging and [^18^F]AlF-NOTA-FAPI-04 PET/CT in differentiating regional lymph node metastases from benign lymph nodes (with *P* value by McNemar *χ^2^* test). The number in the center of each cell is the Chi-square value. The symbols in the top right corners represent *P* values. ns represents no significant difference, * represents *P* < 0.05, ** represents *P* < 0.01, *** represents *P* < 0.001, **** represents *P* < 0.0001.

In the visual analysis, [^18^F]AlF-NOTA-FAPI-04 PET/CT identified 12 false positive lymph nodes. PET/CT visual analysis failed to detect 12 micrometastases in four patients. These findings accounted for the sensitivity and specificity of 95.95% (284/296) and 97.60% (489/501) based on visual analysis. There were significantly higher uptakes of [^18^F]AlF-NOTA-FAPI-04 in regional LNM compared with BLN (*P* < 0.001) (**Table 4**, **Figure 3b**). ROC curve analysis indicated that all parameters were diagnostic indicators for LNM (*P* < 0.001) (**Table 5**, **Figure 4b**). The comparison of diagnostic sensitivity, specificity, and accuracy between conventional imaging and PET/CT was performed using the McNemar *χ^2^* test (**Figure 5**). Conventional imaging showed significantly lower sensitivity (73.99%) than visual analysis (95.95%), SUV_max_ (96.28%), LBR_blood_ (96.62%), and LBR_breast_ (90.20%) (all P < 0.0001). The diagnostic accuracy of conventional imaging (87.45%) was also significantly lower than that of visual analysis (96.99%), SUV_max_ (97.37%), LBR_blood_ (96.74%), and LBR_breast_ (91.34%) (all P < 0.0001). Although the diagnostic specificity of conventional imaging was high (95.41%), except for LBR_breast_, the specificity of conventional imaging in diagnosing LNM was still significantly lower than that of PET/CT (*P* < 0.05).

**Table 5:** ROC quantitative analysis of conventional imaging and [^18^F]AlF-NOTA-FAPI-04 PET/CT in differentiating regional lymph node metastases from benign lymph nodes.

Variables	Cutoff	AUC (95% CI)	*P*	Sensitivity (%)	Specificity (%)	PPV (%)	NPV (%)	Accuracy (%)
Conventional imaging	NA	0.847 (0.815-0.879)	<0.001	73.99	95.41	90.50	86.13	87.45
Visual analysis	NA	0.968 (0.953-0.983)	<0.001	95.95	97.60	95.95	97.60	96.99
SUV_max_	2.14	0.993 (0.989-0.997)	<0.001	96.28	98.00	96.61	97.81	97.37
LBR_blood_	1.85	0.991 (0.985-0.997)	<0.001	96.62	96.81	94.70	97.98	96.74
LBR_breast_	2.68	0.958 (0.944-0.972)	<0.001	90.20	92.02	86.97	94.08	91.34

ROC , receiver operating characteristic; AUC, area under the curve; PPV, positive predictive value; NPV, negative predictive value; NA, not available; SUV, standardized uptake value; LBR, lesion-to-background ratio.

In Level I and II axillary lymph nodes, the sensitivity, specificity, and accuracy of PET/CT visual analysis were all superior to conventional imaging (*P* < 0.05). In Level III axillary and extra-axillary regional lymph nodes, they both had a specificity of 100%. However, the sensitivity and accuracy of conventional imaging were lower than those of PET/CT visual analysis (*P* < 0.001) (**Table 6**).

**Table 6:** Comparisons of diagnostic sensitivity, specificity, and accuracy from conventional imaging and [^18^F]AlF-NOTA-FAPI-04 PET/CT semiquantitative parameters in level I and II axillary lymph nodes, as well as in unsuspected level III axillary and extraaxillary regional lymph nodes.

Diagnostic metric	Level I and II axillary lymph nodes			*P*	Level III axillary and extraaxillary regional lymph nodes		*P*
	Conventional imaging	PET/CT visual analysis			Conventional imaging	PET/CT visual analysis	
Sensitivity (%)	76.52	96.36	<0.001		61.22	93.88	<0.001
Specificity (%)	95.11	97.45	0.022		100.00	100.00	1.000
Accuracy (%)	88.70	97.07	<0.001		76.25	96.25	<0.001

### 3.4 Detection of distant metastases

Although, it is impossible for breast ultrasonography, mammography, and MRI to diagnose distant metastases, all patients underwent additional whole-body CT and bone scan. Therefore, a comparison can be made between the diagnostic performance of PET/CT visual analysis and conventional imaging for distant metastases. 94 distant metastases in 4 patients were detected on [^18^F]AlF-NOTA-FAPI-04 PET/CT images. One liver metastasis was confirmed by biopsy, while all remaining distant metastases were confirmed by follow-up. Bone was the most common metastatic site. Because the liver shows relatively low background activity and physiological clearance of the tracer, PET/CT could accurately detect every liver metastasis (**Figure 2c**). Visual analysis showed that the sensitivity and specificity of [^18^F]AlF-NOTA-FAPI-04 PET/CT were 100%.

**Figure 6:**
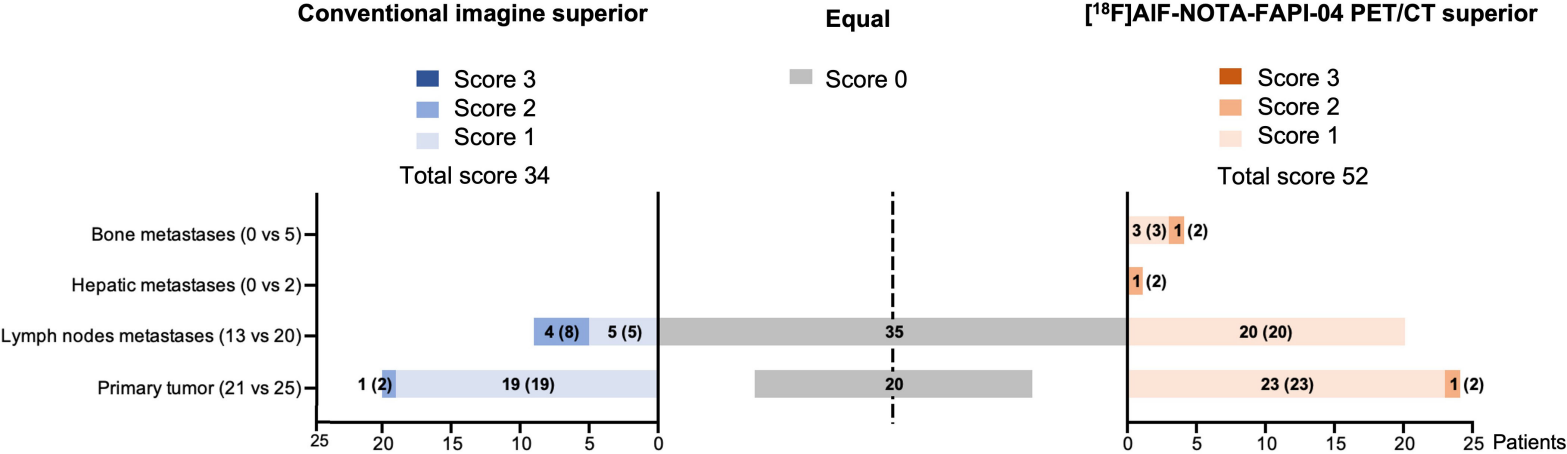
Comparison of visual assessment between [^18^F]AlF-NOTA-FAPI-04 PET and conventional imaging. n(scores) in each bar refers to patient number and total scores.

Whether it was the primary tumor or metastasis, the lesion detectability of [^18^F]AlF-NOTA-FAPI-04 PET/CT was superior to that of conventional imaging, with a much higher score (**Figure 6**).

### 3.5 Accuracy of staging

Compared with conventional imaging, [^18^F]AlF-NOTA-FAPI-04 PET/CT showed higher accuracy in predicting pathological N staging (91.43% *vs.* 65.71%, *P* = 0.008) and final staging (74.29% *vs.* 45.71%, *P* = 0.009) (**Table 7**).

**Table 7:** Diagnostic accuracy and number of misdiagnoses of conventional imaging and [^18^F]AlF-NOTA-FAPI-04 PET/CT.

Staging	Clinical staging (n = 64)			Pathological staging (n = 35)		
	Conventional imaging	PET/CT	*P*	Conventional imaging	PET/CT	*P*
T staging			0.606			0.114
Accuracy	90.63%	85.94%		62.86%	80.00%	
Misdiagnoses	9.37%	14.06%		37.14%	20.00%	
Overestimated	0	0		9	6	
Underestimated	6	9		4	1	
N staging			0.099			0.008
Accuracy	76.56%	89.06%		65.71%	91.43%	
Misdiagnoses	23.44%	10.94%		34.29%	8.57%	
Overestimated	3	3		3	0	
Underestimated	12	4		9	3	
M staging			1.000			1.000
Accuracy	100.00%	100.00%		100.00%	100.00%	
Misdiagnoses	0.00%	0.00%		0.00%	0.00%	
Overestimated	0	0		0	0	
Underestimated	0	0		0	0	
Final staging			0.078			0.009
Accuracy	70.31%	85.94%		45.71%	74.29%	
Misdiagnoses	29.69%	14.06%		54.29%	25.71%	
Overestimated	3	1		8	5	
Underestimated	16	8		11	4	

T, tumor; N, regional lymph node; M, distant metastases.

### 3.6 Comparison between uptake and molecular subtypes in primary lesions

**Figure 7:**
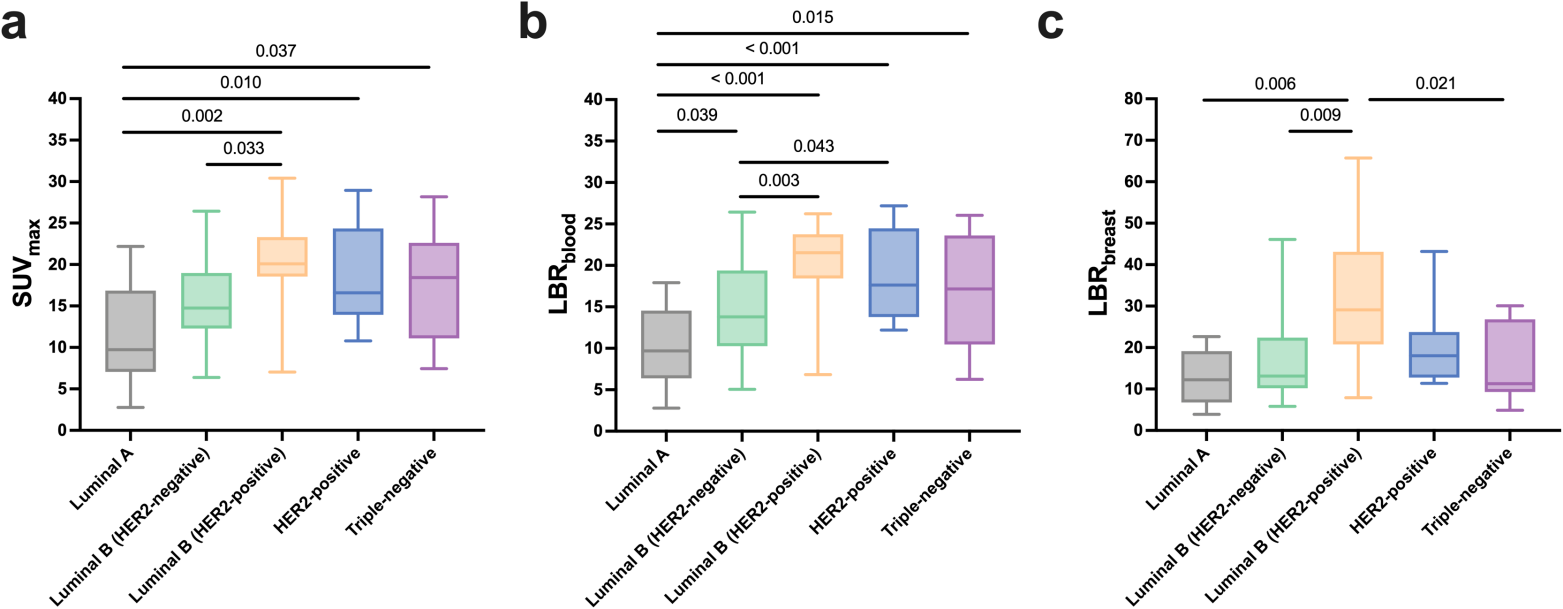
Comparison of semiquantitative [^18^F]AlF-NOTA-FAPI-04 PET/CT parameters among molecular subtypes of primary breast cancer. **(a)** SUV_max_, **(b)** LBR_blood_, and **(c)** LBR_breast_ in luminal A, luminal B (HER2-negative), luminal B (HER2-positive), HER2-positive, and triple-negative breast cancers. Box plots indicate the median, interquartile range, and minimum-to-maximum values. Horizontal lines represent pairwise comparisons, with *P* values shown above.

**Figure 7** shows that luminal A and luminal B (HER2-negative) subtypes had relatively low [^18^F]AlF-NOTA-FAPI-04 uptake, while luminal B (HER2-positive), HER2-positive, and triple-negative subtypes had relatively high uptake.

## 4 Discussion

Although conventional imaging are widely used in BC, their inaccurate qualitative assessments of primary lesions and lower rate of LNM detection strongly influence patients’ staging and treatment decisions. This study is the first *post hoc* retrospective analysis of a prospective trial to evaluate the diagnostic and staging value of [^18^F]AlF-NOTA-FAPI-04 PET/CT in BC. Our key findings include: (1) LBR_breast_ showed excellent discrimination between BC and BBL, but no significant advantage over conventional imaging, whereas PET/CT visual analysis was inferior to conventional imaging. (2) [^18^F]AlF-NOTA-FAPI-04 PET/CT demonstrated significantly superior diagnostic performance with high sensitivity, specificity, and accuracy in detecting regional LNM compared with standard conventional imaging (P < 0.05). (3) [^18^F]AlF-NOTA-FAPI-04 PET/CT demonstrated superior accuracy in predicting pathological staging of BC compared with conventional methods. (4) The uptake of [^18^F]AlF-NOTA-FAPI-04 correlated with the molecular subtypes of BC.

In diagnosing primary breast lesions, visual analysis is a relatively straightforward PET/CT assessment method. The sensitivity and specificity of [^18^F]AlF-NOTA-FAPI-04 PET/CT visual analysis were 98.44% and 70.59%, respectively, which is similar to [^68^Ga]Ga-DOTA-FAPI-04 PET/CT (14, 27) and higher than [^18^F]FDG PET/CT results (28). All false-positive results were observed in premenopausal patients. The breast is a hormone-sensitive organ. Breast density, composed of fibroglandular tissue, can be altered by stimulating endogenous and exogenous hormones. Elevated estrogen levels can lead to an up-regulation of FAP, resulting in increased uptake of [^18^F]AlF-NOTA-FAPI-04 in breast tissue and leading to false positive (29). Therefore, the occurrence of five false positive may be related to the diffused and enhanced FAP expression in the breast tissue, which is influenced by the hormone levels of patients. [^18^F]AlF-NOTA-FAPI-04 PET/CT should be avoided during ovulation (30) or estrogen treatment (31). Thus, the background uptake of breast lesions is essential for BC diagnosis. At a cut-off of 3.86, the sensitivity and specificity of LBR_breast_ were 100% for differentiating BC from BBL, which is the highest record up to now. The essential reason for the 100% diagnostic accuracy is that LBR_breast_ counteracts the influence of varying hormone levels among different patients. This threshold was derived from the ROC analysis. Further validation studies will be necessary to establish this threshold as a robust predictor of test positivity. In the future, the 100% accuracy could even challenge the status of breast biopsy, helping patients exempted from invasive biopsy. But the statement pertains specifically to benign lesions, biopsy is still required for malignant lesions to determine tumor biomarkers.

In detecting regional LNM, the sensitivity (95.95%) and specificity (97.60%) of [^18^F]AlF-NOTA-FAPI-04 PET/CT visual analysis were superior to those of [^68^Ga]Ga-DOTA-FAPI-04 PET/CT (14, 27). Some lymph node micrometastases (< 2mm diameter) in the axillary also failed to be detected. Reactive enlargement of axillary nodes after biopsy could display a challenge for imaging (32). Previous meta-analyses have shown that the sensitivity of ultrasonography for detecting axillary LNM in BC is 51% (95% CI: 43–59%), and the specificity is 100% (95% CI: 99–100%) (5). Mammography has lower sensitivity (20-30%) for detecting axillary LNM (6). [^18^F]AlF-NOTA-FAPI-04 PET/CT can improve sensitivity while maintaining a high specificity and facilitate more accurate N staging. Correctly identifying patients with N0 can avoid unnecessary neoadjuvant treatment, axillary lymph node dissection, and postoperative radiation therapy, which is crucial for improving patients’ quality of life (14). The excellent sensitivity of [^18^F]AlF-NOTA-FAPI-04 PET/CT in Level III axillary and extra-axillary regional lymph nodes aids in determining the extent of lymph node dissection during surgery.

Compared with conventional imaging, [^18^F]AlF-NOTA-FAPI-04 PET/CT showed higher accuracy in predicting pathological N staging (91.43% *vs.* 65.71%, *P* = 0.008), and final staging (74.29% *vs.* 45.71%, *P* = 0.009). However, clinical N staging is enormously influenced by physical examinations, imaging results, and axillary lymph node biopsy findings and is often contradictory to pathological N staging (33). Pathological N staging determines the need for adjuvant chemotherapy, targeted therapy, and radiotherapy. Improved staging accuracy with [^18^F]AlF-NOTA-FAPI-04 PET/CT may have potential implications for perioperative treatment planning, particularly with respect to neoadjuvant therapy selection, extent of axillary surgery, and radiotherapy strategy. However, the actual impact on therapeutic intent was not formally assessed in this retrospective analysis and warrants prospective evaluation. It is important to note that pathological stage analysis was conducted only on patients who underwent surgery directly without receiving neoadjuvant therapy, in this study. For patients receiving neoadjuvant therapy, a pre-operative [^18^F]AlF-NOTA-FAPI-04 PET/CT scan is essential to predict ypT and ypN staging accurately.

We also investigated associations between [^18^F]AlF-NOTA-FAPI-04 uptake and clinicopathologic characteristics of primary BC. We observed statistically significant differences in tracer uptake among molecular subtypes, aligns with the previous results (22). Furthermore, the potential therapeutic applications of FAPI open doors for developing integrated diagnostic and therapeutic probes, promising a new era in personalized BC care (27).

Despite these promising results, our study has limitations. Firstly, we did not directly compare [^18^F]AlF-NOTA-FAPI-04 PET/CT with common [^18^F]FDG PET/CT. Secondly, ethical considerations prevented histological confirmation of all lymph nodes and metastatic lesions, potentially affecting result accuracy. Thirdly, the breast lesions were histopathologically heterogeneous, including different benign and malignant types, which may have leaded to variability in imaging characteristics and diagnostic performance across lesion types. Therefore, the results should be interpreted with caution, particularly when extrapolating the findings to specific histologic subgroups. In addition, although ROC analysis provided valuable insights into the diagnostic performance of the imaging techniques, we acknowledge that the small sample size of 69 patients presents a limitation. Meanwhile, the recruitment of benign and malignant breast lesions was not fully balanced, and the retrospective design limited our ability to control for potential confounding factors. Future studies should use more balanced selection and categorization strategies and consider methods such as propensity score matching to better control for confounders. Finally, this was a monocentric study, which may limit the external validity and generalizability of the findings to other institutions and patient populations.

## 5 Conclusion

Compared with conventional imaging, [^18^F]AlF-NOTA-FAPI-04 PET/CT demonstrated superior diagnostic efficacy in distinguishing benign from malignant breast lesions and in staging BC. In particular, LBR_breast_ (cut-off = 3.86) achieved 100% diagnostic accuracy for primary lesions. Furthermore, [^18^F]AlF-NOTA-FAPI-04 PET/CT significantly improved sensitivity and specificity in detecting regional LNM. Comparing with the conventional imaging, [^18^F]AlF-NOTA-FAPI-04 PET/CT enables more precise prediction of pathological staging and the optimization of perioperative treatment decisions.

## Data Availability

The original contributions presented in the study are included in the article/supplementary material. Further inquiries can be directed to the corresponding authors.
